# Significance of Scapular Fracture Existence in Blunt Chest Trauma: A Retrospective Cohort Study

**DOI:** 10.1155/2024/3550087

**Published:** 2024-05-20

**Authors:** Ashraf F. Hefny, Nirmin A. Mansour, Mohamed A. Hefny, Emad Masuadi, Shadi Al Bahri, Ashraf A. Elkamhawy, Khalid S. Saber

**Affiliations:** ^1^Department of Surgery, College of Medicine and Health Sciences, UAE University, Al Ain, UAE; ^2^Ambulatory Health Services, Abu Dhabi Health Services Company, Abu Dhabi, UAE; ^3^Department of Surgery, Faculty of Medicine, Ain Shams University, Cairo, Egypt; ^4^Department of Public Health, College of Medicine and Health Sciences, UAE University, Al Ain, UAE; ^5^Intensive Care Unit, Sheikh Khalifa Medical City, Abu Dhabi, UAE; ^6^Emergency Department, Sheikh Khalifa Medical City, Abu Dhabi, UAE

## Abstract

**Background:**

Scapular fracture is a rare encounter in blunt trauma patients. The scapula is surrounded by strong groups of muscles offering good protection for the bone. Therefore, a high-energy trauma is needed to cause a scapular fracture. We aim to study scapular fractures and their relation to injury severity and mortality in blunt chest trauma (BCT) patients.

**Methods:**

We retrospectively collected data from all patients with BCT who were admitted to our hospital from December 2014 through January 2017. The injury details of all BCT patients were retrieved from the trauma registry of the hospital and were supplemented by patients' electronic files for missing information. Collected data included demography, mechanism of injury, vital signs, Glasgow Coma Score (GCS) on admission, injured body regions, management, Injury Severity Score (ISS), New Injury Severity Score (NISS), length of hospital stay (LOS), and mortality.

**Results:**

During the study period, 669 patients had BCT. Scapular fracture was present in 29 (4.3%) of the BCT patients. The scapular fracture was missed by chest X-ray in 35.7% of the patients; however, it was accurately diagnosed by computed tomography (CT) scan of the chest. Neck injury was significantly higher in patients with scapular fracture compared with patients without fracture (*p* < 0.001). ISS and NISS were significantly higher in patients with scapular fractures compared to other patients without fractures (*p*=0.04 and *p*=0.003 Mann–Whitney *U* test, respectively). Two patients with scapular fractures died due to severe associated injuries (the overall mortality was 9.6%).

**Conclusions:**

Scapular fracture in BCT patients indicates a high-energy type of trauma. Compared to a chest X-ray, CT scan was more accurate for the diagnosis of scapular fracture. Associated injuries are the main cause of trauma-related mortality rather than the direct effect of the fractured scapula. Particular attention and meticulous evaluation should be paid to head and neck injuries to avoid missing injuries.

## 1. Introduction

Scapular fracture is a rare encounter in blunt trauma patients (0.8%–2%) [1, 2]. The scapula is surrounded by strong groups of muscles offering good protection for the bone that needs high-energy trauma to be fractured [3]. Scapular fractures are usually associated with thoracic injuries such as rib fractures and lung contusions; however, isolated scapular fractures and associated extrathoracic injuries are less common [4, 5].

Although scapular fracture can be diagnosed by using various angles in X-rays, it is best diagnosed with a computerized tomography (CT) scan [3]. CT scan is useful also in the diagnosis of concomitant injuries and helps in guiding the clinical management of blunt chest trauma (BCT) [5].

Scapular fractures are classified anatomically according to the fracture site on the scapula into fracture fossa, body, and processes. However, such classification has no proven prognostic value [6]. Scapular fracture is usually treated conservatively; however, joint stability and neurovascular injury are the main factors for operative intervention [6, 7].

As a part of the shoulder skeleton, many studies in literature have been conducted on the management of scapular fractures from the orthopedic point of view. However, there is a lack of research regarding the importance of the presence of scapular fractures in BCT patients [3, 4].

We aimed to study scapular fractures and their relation to injury severity and mortality in BCT patients.

## 2. Patients and Methods

Data of all patients who were admitted to Al Ain Hospital with BCT were retrospectively collected from December 2014 through January 2017.

The injury details of all BCT patients were retrieved from the Al Ain Hospital trauma registry. The trauma registry of Al Ain Hospital includes all admitted trauma patients. The data in the registry were prospectively collected. All trauma patients or their caregivers sign a consent form accepting the use of their data for auditing and research purposes. Our trauma registry complies with the American College of Surgeons Committee on Trauma National Trauma Data Bank.

In the current retrospective study, we included all patients in the trauma registry with blunt trauma and chest injuries. All other patients with penetrating trauma or patients without chest trauma were excluded. Missed information in the trauma registry were retrieved manually from patients' electronic medical records. The data were collected on a specially designed collection form.

Data included demography, mechanism of injury, vital signs, Glasgow Coma Score (GCS) on admission, injured body regions, management, Injury Severity Score (ISS), New Injury Severity Score (NISS), and outcome including length of hospital stay (LOS), and mortality. Injured body regions include injury of soft tissues, neurovascular, organs, and/or bones in each region. Spine injury includes all the vertebrae in the body.

The main objectives were to study the management of scapular fractures in BCT patients and their relation to injury severity and mortality. Ethical approval for this study was obtained from the Tawam Human Research Ethics Committee, Abu Dhabi, UAE (T-HREC Ref. MF2058-2023-952).

A comparison between BCT patients who sustained scapular fracture and other patients without fracture was performed. Statistical analyses were performed using the Statistical Package for the Social Sciences (IBM SPSS Version 28, Chicago, II, USA). Data are presented as the mean (standard deviation), median (range), or number (%), as appropriate. The chi-square test was used for categorical data in comparisons between groups. For continuous or ordinal data, the Mann–Whitney *U* test was used to compare two independent groups, whereas Fisher's exact test was used to compare two independent groups for categorical data. A *p* value of ≤0.05 was considered statistically significant.

## 3. Results

During the study period, there were 4779 patients included in the trauma registry of Al-Ain Hospital, and 669 (13.9%) patients had blunt chest trauma. Scapular fracture was present in 29 (4.3%) of the BCT patients, all were males except one female patient. The median (range) age of patients with scapular fracture was 29 (1–86) years, which was not statistically different from other patients without fractures (*p*=0.264, Fisher's exact test).

5 (17.2%) UAE national patients and 24 (82.8%) non-UAE national patients had scapular fractures. Bangladeshi nationality patients were the most commonly injured nationality with scapular fractures followed by Pakistani patients in 8 (27.6%) and 7 (24.1%) patients, respectively (Table 1).

Road traffic collision (RTC) was the most common mechanism of blunt chest injury in 17 (58%) patients with scapular fracture, followed by falls from a height of more than one meter and falling of heavy objects in 6 (20.7%) and 3 (10.3%) patients, respectively.

In the current study, work-related trauma occurred in 118 (17.6%) patients with BCT. However, scapular fracture was significantly higher in work-related injury in 10 (34.5%) patients compared to 108 (17.7%) patients without scapula fracture (*p*=0.024 Fisher's exact test).

Chest X-ray was performed in 28 patients with scapula fracture before chest CT scan. Scapular fracture was not detected on the chest X-ray of 10 (35.7%) of those patients.

The right scapula was fractured in 13 (44.8%) patients, the left was fractured in 14 (48.3%) patients, and 2 (6.9%) patients had bilateral scapular fractures.

There was no statistically significant difference in cardiac enzyme levels (creatine kinase, creatine kinase MB, and troponin) between patients with scapular fracture and patients without fracture (*p*=0.585, *p*=0.185, and *p*=0.391, respectively).

ISS and NISS were significantly higher in patients with scapular fractures compared to other patients without fractures (*p*=0.04 and *p*=0.003 Mann–Whitney *U* test, respectively).

Table 2 shows a comparison between different variables in BCT patients with scapular fractures and other patients without scapular fractures.

**Figure 1:**
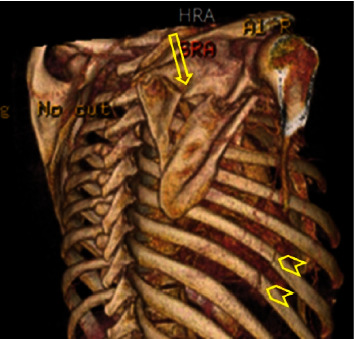
34-year-old male, unrestrained backseat passenger, was involved in a rollover car collision and he was ejected from the car. CT scan chest showed a comminuted displaced fracture of the right scapula (arrow) and undisplaced fractures of the right lateral sixth and seventh ribs (arrowheads).

Pneumothorax, lung contusion, multiple rib fractures, and fractures of the first and/or second ribs were significantly associated with scapular fracture (*p*=0.020, *p*=0.029, *p*=0.012, and *p* < 0.001 Chi-Square test, respectively) (Table 3) (Figure 1).

Neck injuries which include neurovascular and soft tissue injuries (vertebral injury included in the spinal region) were significantly higher in 17 (58.6%) patients with scapular fracture compared to 115 (18%) patients without fracture (*p* < 0.001) (Table 4).

The scapular fractures in the current study were treated conservatively in all patients.

Two patients with scapular fractures died (the overall mortality was 9.6%) (Table 2). One of the deceased patients was a front-seat passenger who was involved in RTC and sustained severe traumatic brain injury (GCS of 3) and left scapular fracture on a portable X-ray chest. The patient was in a hemorrhagic shock and focused assessment with sonography (FAST) showed intraperitoneal bleeding due to liver laceration. The patient died during the resuscitation process.

The other patient was involved in a rollover RTC. She had severe traumatic brain injury (GCS of 3) with fixed dilated pupils bilaterally, bilateral lung contusion, and scapular fracture. She died in the ICU on day 13 secondary to multiorgan failure.

## 4. Discussion

The scapular fracture is not easily identified on chest X-ray radiography [5]. In the current study, the diagnosis of scapular fracture was missed in 35.7% of the patients who had chest X-rays; however, they were accurately diagnosed on a CT scan of the chest. Historically, certain angles and patient positions were used to increase the likelihood of diagnosing scapular fractures on X-ray radiography, which is difficult to apply in the trauma setting [8, 9]. The introduction of CT scan as a part of the early trauma management protocol in recent years has led to a more accurate diagnosis of scapular fractures [5, 8, 10]. A 10-year retrospective study of the National Trauma Data Bank in the United States showed an increased number of scapular fractures diagnosed corresponding to the introduction of CT as a part of the trauma protocol [3]. CT scan has high capability in image processing to view different windows with or without three-dimensional reconstruction [5]. Moreover, CT scan helps to guide the management plan of injured patients by diagnosing the concomitant injuries associated with the scapular fracture [5].

The majority of injured patients (82%) with scapular fractures in our study were non-UAE nationals who constitute the majority of the population (78%) in UAE [11]. Most of those injured patients were young adult males who were low-income workers from the Indian subcontinent (Bangladesh, Pakistan, and Afghanistan). The fast economic growth in the UAE was associated with an increased number of foreign laborers working in major infrastructure projects. Many of those laborers are using cheap ways of transportation to their work such as going on foot or using bicycles without using protective gear which may expose them to serious RTC [12, 13]. Also, many of them are working on construction sites or as drivers without proper protective equipment [14, 15]. In a previous study, occupational injuries accounted for about 30% of trauma injuries in the UAE [15]. The current study showed that scapular fracture was significantly higher in patients with work-related injuries compared to other patients without scapular fractures. This can be related to the higher energy impact of such trauma on workers who may not have protective equipment [15]. Effective countermeasures have been taken by the UAE to reduce the incidence and severity of occupational injury among vulnerable workers such as legislation for compulsory wearing of protective gear in workplaces and helmets for bicycle users [16]. Ensuring a safe work environment and other preventive measures should be effectively implemented to reduce the incidence of work-related injury. Many of those injured non-UAE nationals were followed up for a short period because most of them preferred to return to their home country whenever they could after apparent recovery [17].

In agreement with previously published studies, the main mechanism of injury was RTC followed by a fall from a height [1, 3, 18]. There was no significant difference regarding these mechanisms between BCT patients with scapular fractures and other patients without fractures.

Two patients had bilateral scapular fractures which are rare in trauma patients. However, most of these cases in the literature resulted from electric shock or epileptic convulsions [19, 20].

A previously published cross-sectional study on scapular fracture with or without BCT showed no significant difference in vital signs between patients with and without scapular fracture [1]. However, the current study which was conducted on BCT patients showed that hemodynamic parameters including vital signs and the need for blood transfusion were significantly different between patients with scapular fracture and other patients without fractures (Table 2). This may reflect the severity of injuries in patients with scapular fracture in BCT patients and the need for close observation of vital signs and hemodynamic status of BCT patients with scapular fracture.

In agreement with other studies, scapular fracture was significantly associated with thoracic injuries such as multiple rib fractures and pneumothorax [1, 2, 4] (Table 3). A retrospective study over 6 years conducted in the United Kingdom showed that there was no significant difference regarding lung contusion between patients with scapular fractures and patients without fractures [18]. However, the current study showed that lung contusion was significantly higher in patients with scapular fractures. This difference can be explained by the increased trend of chest CT scan usage as a screening tool in the trauma settings which diagnoses lung contusion more accurately than the usual chest X-ray [5]. Overall, thoracic injuries are severe and more frequent in patients with scapular fracture, which reflects the higher injury applied during the impact leading to scapular fracture.

Similar to other studies, BCT patients with scapular fractures have suffered from associated extrathoracic injuries in different body regions (Table 3). Abd El-Shafy et al. showed that the scapular fracture was associated with great vessel injury, mainly the carotid artery in the neck region [21]. Another study showed that vertebral fracture, especially in the neck region, was significantly related to the scapular fracture in BCT [4]. The current study showed that the neck region was the most common extrathoracic injured body region and scapular fracture was significantly associated with neck injury compared to other patients without fracture. This may be related to the proximity of the scapula to the neck region. The clinician should have a higher index of suspicion to avoid missing the diagnosis of neck injury, especially in patients with altered level of consciousness.

Similar to other studies, there was no significant difference in GCS between patients with scapular fracture and others without fracture as it depends more on head injury rather than thoracic injury [1, 18].

Injury severity parameters such as ISS, NIS, and ICU admission were significantly higher in patients with scapular fracture compared to other patients without fracture, which is similar to other studies [1, 4, 18]. Accordingly, as expected, the length of hospital stay was significantly longer in patients with scapular fracture patients who had more severe injuries.

Scapular fracture was treated conservatively in all patients in this study, which is similar to another study [22]. Two patients had bilateral scapular fractures which are rare in trauma patients. These were cases with severe direct high-energy impact on the thoracic cage. One of them had a heavy object falling on his chest at the workplace and the other patient had severe RTC.

In agreement with other studies, there was no significant difference in mortality which was related to other associated injuries (mainly head injury) [2, 18].

The study is a retrospective single-center study with a relatively small sample size. Also, the retrospective nature of this study precluded the collection of missed injuries which were not included in the trauma registry data and could not be identified in patients' files. A prospective study with more details on missing injuries and their effect on the outcome would be of great value. However, the current study has an important impact on the evaluation of BCT patients with scapular fractures and helps to define the prognostic factors that will help in improving the management plan of those patients.

## 5. Conclusions

Scapular fracture in blunt chest trauma patients indicates a high-energy type of trauma. Compared to chest X-ray, CT scan is more accurate for the diagnosis of scapular fracture. Close observation of vital signs and hemodynamic status of BCT patients with scapular fractures is essential to avoid any deterioration of the patients' hemodynamic status. Particular attention and meticulous evaluation should be paid to head and neck injuries to avoid missing injuries. Associated injuries are the main cause of trauma-related mortality rather than the direct effect of the fractured scapula. Enforcement of legislation for a safe work environment and worker protection is essential to prevent work-related injuries.

## Figures and Tables

**Table 1:** The nationality of blunt chest trauma patients with scapular fracture (*n* = 29) and other patients without scapular fracture (*n* = 640).

Nationality	Scapula fracture *N* (%)	No scapula fracture *N* (%)
Bangladesh	8 (27.6%)	72 (11.25%)
Pakistan	7 (24.1%)	149 (23.3%)
United Arab Emirates	5 (17.2%)	136 (21.25%)
Afghanistan	3 (10.3%)	34 (5.3%)
Egypt	2 (6.9%)	36 (5.7%)
India	1 (3.4%)	53 (8.3%)
Oman	1 (3.4%)	32 (5%)
Others	2 (6.9%)	128 (20%)

**Table 2:** A comparison of different variables between blunt chest trauma patients with scapular fracture (*n* = 29) and other patients without scapular fracture (*n* = 640).

Variable	Scapula fracture *N* (%)	No scapula fracture *N* (%)	*p* value^*∗*^
Systolic BP mean (range)	126 (77–161)	134 (47–218)	0.028
Pulse mean (range)	99 (66–178)	89 (4–180)	0.008
Respiratory rate mean (range)	22 (16–45)	20 (10–54)	0.007
O2 saturation mean (range)	97 (81–100)	98 (54–100)	0.975
GCS mean (range)	14 (3–15)	14 (3–15)	0.328
ICU LOS mean (range)	7 (1–13)	11 (1–96)	0.497
ISS mean (range)	15 (5–29)	11 (1–45)	0.003
NISS mean (range)	18 (5–36)	13 (1–50)	0.002
Intubation *n* (%)	5 (17.2%)	50 (7.8%)	0.081
ICU admission *n* (%)	8 (27.6%)	86 (13.4%)	0.049
Chest tube insertion *n* (%)	4 (13.8%)	34 (5.3%)	0.075
Blood transfusion *n* (%)	10 (34.5%)	71 (11.1)	0.001
Hospital LOS mean (range)	8 (1–26)	6 (1–95)	0.029
Mortality *n* (%)	2 (6.9%)	13 (2%)	0.134

^*∗*^ *p* value: Fisher's exact test or the Mann–Whitney *U* test.

**Table 3:** Thoracic injuries in blunt chest trauma patients with scapular fracture (*n* = 29) and other patients without scapular fracture (*n* = 640).

Injury *n* (%)^*∗*^	Scapula fracture *N* (%)	No scapula fracture *N* (%)	*p* value^*∗∗*^
Pneumothorax *n* (%)	12 (41.4%)	145 (22.7%)	0.026
Hemothorax *n* (%)	4 (13.8%)	55 (8.6%)	0.312
Lung contusion *n* (%)	16 (48.3%)	172 (26.9%)	0.046
Multiple ribs # *n* (%)	14 (41.4%)	145 (22.7%)	0.018
Flail chest *n* (%)	1 (3.4%)	6 (0.9%)	0.268
1^st^ and/or 2^nd^ rib # *n* (%)	8 (27.6%)	41 (6.4%)	<0.001
Single rib *n* (%)	3 (10.3%)	51 (8%)	0.722
Sternum # *n* (%)	1 (3.4%)	32 (5%)	1
Heart injury *n* (%)	1 (3.4%)	6 (0.9%)	0.268
Great vessels injury *n* (%)	1 (3.4%)	5 (0.8%)	0.234
Surgical emphysema *n* (%)	3 (10.3%)	32 (5%)	0.189
Soft tissue injury *n* (%)	14 (48.3%)	340 (53.1%)	0.705
Diaphragm injury *n* (%)	0 (0%)	2 (0.3%)	1

^*∗*^The percentage is more than 100% because some patients have more than one injury. ^*∗∗*^*p* value: Fisher's exact test or the Mann–Whitney *U* test.

**Table 4:** Different injured body regions in blunt chest trauma patients with scapular fracture (*n* = 29) and other patients without scapular fracture (*n* = 640).

Region *n* (%)^*∗*^	Scapula fracture *N* (%)	No scapula fracture *N* (%)	*p* value^*∗∗*^
Head *n* (%)	13 (44.8%)	273 (42.7%)	0.849
Spine *n* (%)	7 (24.1%)	136 (21.3%)	0.65
Neck *n* (%)	17 (58.6%)	115 (18%)	<0.001
Abdomen *n* (%)	9 (31%)	125 (19.5%)	0.152
Lower limb *n* (%)	7 (24.1%)	147 (23%)	0.825

^*∗*^The percentage is more than 100% because some patients have more than one injured region. ^*∗∗*^*p* value: Fisher's exact test or the Mann–Whitney *U* test.

## Data Availability

The datasets generated and/or analyzed during the current study are available from the corresponding author upon request.
